# Early neurovascular dysfunction in a transgenic rat model of Alzheimer’s disease

**DOI:** 10.1038/srep46427

**Published:** 2017-04-12

**Authors:** Illsung L. Joo, Aaron Y. Lai, Paolo Bazzigaluppi, Margaret M. Koletar, Adrienne Dorr, Mary E. Brown, Lynsie A. M. Thomason, John G. Sled, JoAnne McLaurin, Bojana Stefanovic

**Affiliations:** 1 Department of Medical Biophysics, University of Toronto, 610 University Avenue, Toronto, Ontario, M5G 2M9, Canada; 2 Sunnybrook Health Sciences Center, 2075 Bayview Avenue, Toronto, Ontario, M4N 3M5, Canada; 3 Fundamental Neurobiology, Krembil Research Institute, University Health Network, 60 Leonard Avenue, Toronto, Ontario, M5T 2R1, Canada; 4 Hospital for Sick Children, 555 University Avenue, Toronto, Ontario, M5G 1X8, Canada; 5 Department of Laboratory Medicine and Pathobiology, University of Toronto, 1 King’s College Circle, Toronto, Ontario, M5S 1A1, Canada

## Abstract

Alzheimer’s disease (AD), pathologically characterized by amyloid-β peptide (Aβ) accumulation, neurofibrillary tangle formation, and neurodegeneration, is thought to involve early-onset neurovascular abnormalities. Hitherto studies on AD-associated neurovascular injury have used animal models that exhibit only a subset of AD-like pathologies and demonstrated some Aβ-dependent vascular dysfunction and destabilization of neuronal network. The present work focuses on the early stage of disease progression and uses TgF344-AD rats that recapitulate a broader repertoire of AD-like pathologies to investigate the cerebrovascular and neuronal network functioning using *in situ* two-photon fluorescence microscopy and laminar array recordings of local field potentials, followed by pathological analyses of vascular wall morphology, tau hyperphosphorylation, and amyloid plaques. Concomitant to widespread amyloid deposition and tau hyperphosphorylation, cerebrovascular reactivity was strongly attenuated in cortical penetrating arterioles and venules of TgF344-AD rats in comparison to those in non-transgenic littermates. Blood flow elevation to hypercapnia was abolished in TgF344-AD rats. Concomitantly, the phase-amplitude coupling of the neuronal network was impaired, evidenced by decreased modulation of theta band phase on gamma band amplitude. These results demonstrate significant neurovascular network dysfunction at an early stage of AD-like pathology. Our study identifies early markers of pathology progression and call for development of combinatorial treatment plans.

Alzheimer’s disease (AD), the most common form of dementia in the elderly, is characterized by progressive cognitive decline, Aβ deposition, neurofibrillary tangle (NFT) formation, and neurodegeneration^1^. AD is also associated with cerebrovascular pathologies, including morphological alterations in the cerebral vasculature^2^,^3^ and blood-brain barrier (BBB) dysfunction^4^. Mural cells, which form the outer layer of cerebral vessels and are responsible for vascular contraction^5^ and structural maintenance of cerebral vessels^6^ and BBB^7^,^8^,^9^,^10^, have been shown to be deficient in AD brains post mortem and associated with breakdown of BBB^7^. Further, a recent study demonstrated the contribution of mural cell degeneration to the progression of AD pathologies: specifically, pericyte-deficient platelet-derived growth factor receptor beta (PDGFRβ) knockout mice^11^,^12^ crossed with transgenic mice overexpressing Swedish mutant of human amyloid precursor protein (APP_sw_) exhibited accelerated progression of Aβ accumulation and cognitive decline^13^. In addition to these morphological changes, there is mounting evidence on the significance of cerebrovascular dysfunction for AD progression^14^,^15^,^16^,^17^,^18^. A recent study by Iturria-Medina and colleagues demonstrated vascular dysfunction to be the earliest abnormality in AD pathology progression^19^. Nonetheless, the etiology and pathophysiology of cerebrovascular impairment and its relation to cognitive decline in AD remain uncertain.

In both humans and rodents, spatial memory representations of the neuronal network are related to temporal modulation of theta oscillations and theta-gamma coupling^20^,^21^. AD patients present a reduced coupling of resting state EEG rhythms among cortical regions^22^,^23^,^24^,^25^, which suggests that coupling of cortical rhythms at specific frequency bands and aberrated cortical EEG coherence may be specific markers of AD. It has been speculated that the impairment in EEG functional coupling is modulated by the cholinergic system and that the decreased cortical EEG coherence results from impaired functioning of basal forebrain cholinergic inputs to the cortex and hippocampus^25^,^26^. Hence, amplitude modulation analysis of resting neuronal oscillations has been suggested as a promising tool to characterize different neurological disorders^27^,^28^ including early stage AD^29^,^30^.

Thus far, the majority of cerebrovascular and neuronal dysfunction associated with AD has been observed in transgenic murine models of AD that manifest age-dependent Aβ accumulation^14^,^16^,^31^,^32^. In comparison, neuropathological studies on triple transgenic mice that exhibit both Aβ and tau pathology showed *accelerated* rate of neuronal compromise and Aβ-independent neuronal loss in the later stage, indicating significant role of tauopathy in the progression of AD^33^,^34^. In addition, a recent study investigating the effect of tau pathology on cerebrovascular function in the absence of Aβ accumulation demonstrated unexpected *increases* in cerebrovascular reactivity to hypercapnia in transgenic rTg4510 mouse model of AD in late stage of tau pathology when compared to that observed in age-matched non-transgenic (nTg) littermates^35^. While Aβ accumulation and NFT formation are both associated with toxicity^36^ and exacerbate degeneration^36^,^37^, their cumulative effect on the cerebrovascular function remains unclear, particularly as they may have opposing effects on the magnitude of cerebrovascular reactivity^35^. Examining the neurovascular dysfunction in the presence of both amyloid and tau pathologies is thus of particular import yet has not been done to date.

To address this gap, we set out to characterize the extent of early cerebrovascular and neuronal network impairment in the TgF344-AD rat model that exhibits progressive amyloidosis, tauopathy, and frank neuronal loss in addition to cognitive decline^38^. Neuronal network functioning was assessed by measuring phase-amplitude coupling (PAC) via linear array electrode recordings. We evaluated Aβ accumulation using immunohistochemistry and tau hyperphosphorylation and morphological alterations of mural cells through Western blot and immunofluorescence. *In situ* two-photon fluorescence microscopy was performed to visualize morphology and function of the cortical microvascular network. Profound cerebral microvascular and neuronal network dysfunction was demonstrated at the very onset of cognitive dysfunction when soluble Aβ species and hyperphosphorylated tau begins to rise in this model^38^.

## Materials and Methods

### Rats

We used male and female TgF344-AD rats on the Fisher 344 background, overexpressing APP_sw_ and Δ exon 9 mutant human presenilin-1 (PS1ΔE9) under mouse prion protein promoter^38^. Age-matched TgF344-AD and their homozygous non-transgenic (nTg) littermates were kept on a 12 hr:12 hr light/dark cycle with water and food *ad libitum*. A total of 54 9-month old animals (28 TgF344-AD and 26 nTg) were employed. All experimental procedures were approved by the Animal Care Committee of the Sunnybrook Health Sciences Center, which adheres to the Policies and Guidelines of the Canadian Council on Animal Care and meets all the requirements of the Provincial Statute of Ontario, Animals for Research Act as well as those of the Federal Health of Animals Act.

### Isolation of vessel-enriched fractions and Western blots

Western blots were carried out on vessel-enriched fractions isolated from dry ice-frozen whole brains^39^ acquired from 28 rats (14 TgF344-AD; 14 nTg). Rats were trans-cardially perfused with phosphate-buffered saline (PBS) before brains were extracted. The tissues were first mechanically homogenized in 0.1 M ammonium carbonate, 5 mM ethylenediaminetetraacetic acid (EDTA), 0.01% sodium azide, and 1% protease inhibitor cocktail (539134, Millipore), then centrifuged at 100,000 g (one hour, 4 °C). Pellets were re-suspended in 0.1 M ammonium carbonate, 7% sodium dodecyl sulphate (SDS), and 2% protease inhibitor cocktail, stirred on ice for four hours, and filtered through 40 μm mesh filters to isolate blood vessel tufts that were in turn sonicated in 10 mM Tris pH 8.0, 1 mM EDTA, 1 mM ethylene glycol tetraacetic acid, 0.8% Triton, 0.2% SDS, 140 mM NaCl, and 1% protease inhibitor cocktail) and centrifuged (17,000 g, 15 minutes, 4 °C). Supernatants were subjected to bicinchoninic acid protein assays (23225, Thermo-Fisher) and electrophoresis. For tau immunoblots, whole brains of PBS-perfused rats were mechanically homogenized and sonicated in 10 mM Tris pH 8.0, 1 mM EDTA, 1 mM ethylene glycol tetraacetic acid, 0.8% Triton, 0.2% SDS, 140 mM NaCl, and 1% protease inhibitor cocktail, centrifuged (17,000 g, 15 minutes, 4 °C), then subjected to bicinchoninic acid protein assays and electrophoresis. Prior to electrophoresis, samples were boiled in lithium dodecyl sulfate sample buffer (NP0007, Thermo-Fisher) containing 1% β-mercaptoethanol. Boiled samples were electrophoresed in 4–12% bis-Tris gels (NP0336, Thermo-Fisher), and transferred to nitrocellulose membranes, which were blocked for one hour in Tris-buffered saline-0.1% Tween (TBS-T) containing 5% milk, probed with primary antibodies (in TBS-T containing 5% milk) overnight at 4 °C, probed for one hour with horseradish peroxidase-conjugated secondary antibodies (in TBS-T containing 5% milk), and then developed using ECL prime (RPN2232, GE Life Sciences). Densities of the Western blots were quantified using ImageJ. We used the following antibodies: anti-desmin (1:100, M0760, Dako), anti-α-smooth muscle actin (α-SMA, 1:1000, ab125025), anti-platelet-derived growth factor receptor-β (PDGFRβ, 1:500, AF1042, R&D), anti-NG2 (1:500, ab5320, Millipore), anti-occludin (1:100, 71–1500, Thermo-Fisher), anti-glial fibrillary acidic protein (GFAP, 1:1000, sc-6170, Santa Cruz), AT8 anti-phosphorylated tau (1:500, MN1020, ThermoFisher), AT180 anti-phosphorylated tau (1:500, MN1040, ThermoFisher), Ser199 anti-phosphorylated tau (1:2000, 29957, Cell Signaling), anti-glyceraldehyde 3-phosphate dehydrogenase (GAPDH, 1:50000, G9545, Sigma), and horseradish peroxidase-conjugated anti-IgG (1:2000, sc-2004/2005/2020, Santa Cruz)^16^.

### Immunofluorescence and immunohistochemistry

Ten rats were trans-cardially fix-perfused with PBS followed by 4% paraformaldehyde (in PBS). Extracted brains were post-fixed overnight in 4% paraformaldehyde (in PBS), washed three times with PBS, and then embedded in 30% sucrose (in PBS) for three days at 4 °C. Sucrose embedded brains were frozen and sectioned coronally at 40 μm/section. Floating sections were first subjected to antigen retrieval through heating at 85 °C in 10 mM sodium citrate (pH 6.0) and 0.1% Tween for 45 minutes. The sections were then stained with 1% ThioS (in water) for 7 minutes and differentiated twice in 70% ethanol (five minutes each). After three washes in PBS, the sections were blocked in PBS containing 0.5% Triton and 5% normal donkey serum for one hour, then probed with primary antibodies (in 0.5% Triton and 5% normal donkey serum, at 4 °C overnight). After another three PBS washes, the sections were simultaneously probed with fluorescence-conjugated secondary antibodies and tomato lectin (in 0.5% Triton and 5% normal donkey serum, for two hours), washed three times in PBS, then mounted on slides. Images of blood vessels were acquired using a Leica TCS SP5 confocal microscope. For quantification of mural cells, three evenly-spaced coronal sections between + 1.50 mm and −0.50 mm relative to bregma were sampled. Images were acquired along the pial surface of the somatosensory cortex. For each animal, 20 images containing 1–3 penetrating vessels (>10 μm in diameter and perpendicular to the pial surface) were included in the analysis. Total pixels in each fluorescent channel were summed after thresholding background pixel density to 7–10 pixels per 150 μm^2^. Mural cell coverage was expressed as total desmin-positive pixels over total lectin-positive pixels. Mural cell detachment was expressed as desmin-positive pixels in detached cells (those not in contact with other desmin-positive cells) over total desmin-positive pixels. Mural cell dedifferentiation was expressed as desmin-positive pixels in dedifferentiating cells over total desmin-positive pixels. Dedifferentiating mural cells in arterioles were defined as desmin-positive cells that extended neither parallel nor perpendicular to the vascular wall. Dedifferentiating mural cells in venules were defined as desmin-positive cells with processes shorter than both the lengths and widths of their cell bodies.

Amyloid-β plaques were quantified using 6F3D labeling. Floating coronal sections of 40-μm thickness were quenched with 3% hydrogen peroxide (in PBS) for 30 minutes before 70% formic acid (in water) retrieval for 7 minutes. The sections were then incubated with the 6F3D antibody (in PBS with 0.25% Triton and 0.2% bovine serum albumin) at room temperature overnight, the bioninylated anti-IgG for 2 hours (in PBS with 0.25% Triton and 0.2% bovine serum albumin, 1:400, PK4002, Vector), and the avidin complex for 1 hour (in PBS, 1:200, PK4002, Vector) before development with diaminobenzidine (SK4100, Vector). Brightfield micrographs (stitched image of the whole section) of 6 sections per brain were acquired with a 4x objective using Stereo Investigator (MBF Bioscience) and analyzed by ImageJ. Aβ plaque load is expressed as total 6F3D pixels over total area of hippocampus or cortex. The threshold range in each section was set such that the smallest plaques (~100 μm^2^ in area) were preserved.

We used the following antibodies: anti-desmin (1:50, M0760, Dako), anti-α-SMA (1:500, ab5694, Abcam), 6F3D (1:400, M0760, Dako), fluorescence-conjugated anti-IgG (1:200, A11003/A31573, Thermo-Fisher), and Texas Red-conjugated tomato lectin (1:200, TL-1176, Vector).

### Two-photon fluorescence microscopy

#### Surgical preparation

Twenty-two 9-month old rats (12 TgF344-AD; 10 nTg) were designated for *in vivo* two-photon fluorescence microscopy. Surgical procedures used were developed based on those employed previously in mice^14^,^16^. The rats were anaesthetized with isoflurane (5% for induction and 2–3% for maintenance) in medical air, supplemented with oxygen to achieve a total concentration of 30–35% oxygen. Proper hydration was ensured with subcutaneous administrations of 2 ml lactated Ringer’s solution every 3 hours. Body temperature was maintained at 37 °C via feedback-controlled heating pad (TC-1000, CWE Inc.). The rats were tracheostomized for mechanical ventilation (SAR 830/P, CWE Inc.), and the composition of inhaled gas over time was prescribed by programming a gas mixer (GSM-3, CWE Inc.). Animals were secured on the stereotaxic frame with ear and incisor bars. A circular craniotomy 5-mm in diameter was centered at AP −3.0 mm and ML ± 2.5 mm over the parietal region. After removing the dura, the cranial window was closed with an 8-mm circular glass coverslip (World Precision Instruments) secured to the skull with cyanoacrylate adhesive. A well was created with dental cement (Ortho-jet Liquid, Lang Dental) around the cranial window, which was filled with double-distilled water for the water immersion objective (25x, NA 1.05; Olympus Corp., Japan). To enable cerebral blood flow measurements during normocapnia and hypercapnia, a tail vein catheter was implanted for multiple injections of 70 kDa Texas Red (Invitrogen) fluorescent dextran boluses (7 mg/kg each bolus; dissolved in PBS). Motion due to respiratory and cardiac cycles was minimized by intravenous injection of pancuronium bromide (1 mg/kg every hour, Sigma-Aldrich).

To allow *in vivo* imaging of amyloid load^40^, Methoxy-X04 (12 mg/kg, Tocris) was injected intraperitoneally under isoflurane twenty-four hours prior to the imaging of each TgF344-AD rat. Two TgF344-AD and one nTg rat did not survive the surgical preparation due to surgery-associated complications.

#### Data acquisition

Imaging was performed on an FV1000MPE multiphoton laser scanning microscope (Olympus Corp., Japan), equipped with a Ti:Sapphire tunable laser (Mai Tai HP; 690–1040 nm; Newport Corp.). Texas Red and Methoxy X-04 were excited at 910 nm and 780 nm, respectively. The resulting fluorescence emission was collected with external photomultiplier tubes (Hamamatsu) preceded by a 575–630 nm bandpass filter for Texas Red, and a 420–460 nm bandpass filter for Methoxy-X04.

A 512 μm × 512 μm area parallel to the cortical surface was imaged at a cortical depth of 150–200 μm, with nominal in-plane resolution of 0.5 μm × 0.5 μm. Based on this acquisition, free-hand line scans (10 μm/pixel, 20 ms/line, ~600 lines) were collected over multiple cortical penetrating vessels (preferentially arterioles) and any capillaries found along the trajectory of the scan. The start of the line scan acquisition triggered the injection of the fluorescent dextran via a syringe pump: microvascular transit times were thus estimated in relation to the time of the bolus injection. Free-hand line scans lasting 11–13 seconds were performed to capture the passage of the bolus in the select vessels during periods of normocapnia (0% FiCO_2_), alternating with brief (ca. 60 s) periods of hypercapnia (10% FiCO_2_), where the FiCO_2_ was controlled by a pre-programmed gas mixer. Up to six bolus injections were performed at three different cortical locations in each rat. Thereafter, the cortical microvascular architecture and Aβ deposits were imaged by acquiring a series of 133–300 slices parallel to the cortical surface at 4 μs/pixel, 512 μm × 512 μm, with nominal in-plane resolution of 1 μm × 1 μm, every 1.5 μm, to a depth of 200–450 μm below the cortical surface. Bolus tracking data from 3 TgF344-AD rats could not be analyzed due to abrupt changes in imaging plane from motion during hypercapnia and imaging quality degradation during the scanning.

#### Bolus tracking data analysis

Maximum intensity projection, along the time dimension, of the line scan data was taken to identify the vessels. The bolus passage, during both normocapnia and hypercapnia, was modeled using the gamma variate function^41^,^42^, as described previously^14^,^16^ to estimate the time-to-peak (TTP), which represents the time interval between the microscope-triggered injection of the bolus by the pump and the time of peak fluorescent signal intensity in the vessel. TTP during CO_2_ challenge was modeled as a linear function of the TTP during air breathing, using orthogonal regression (deming function in deming package, R) to account for errors in TTP measurements in either state. The flow change was then estimated by the inverse of the slope of this regression line, thus assuming decreased dispersion of time-to-peak during hypercapnia relative to normocapnia^43^.

#### Morphological data analysis

Imaris software (Bitplane Inc.) was employed for semi-automatic intensity-based segmentation of cortical microvascular network and vascular amyloid on cortical penetrating vessels. The vascular amyloid load of each cortical penetrating vessel was calculated as the ratio of the amyloid surface area to the surface area of the vessel. The penetrating vessels were classified as arterioles or venules based on their morphological features^44^,^45^. Vessels were first classified into pial arteries and veins after surgical preparation of the cranial window: light red pial vessels exhibiting smaller diameter and fewer branches were designated as arteries, whereas those vessels with dark red blood exhibiting larger diameter and more branches were identified as veins. This pial vessel classification was used to determine which penetrating vessels to select for the free-hand line scan. The classification of a penetrating vessel as an arteriole or venule was then determined through analysis of the 3D cortical scans, tracing back the connections between cortical penetrating vessels and their parent pial vessels. These designations were then confirmed based on the morphological characteristics of cortical penetrating vessels: penetrating arterioles exhibit fewer branches and maintain a constant diameter throughout the cortex, whereas penetrating venules exhibit more branching, and variable diameter as they traverse the cortical depth. Finally, these designations were validated using the bolus-tracking data, assuming delayed bolus arrival in venules relative to the arterioles. Any vessels less than 10 μm in apparent diameter were deemed capillaries.

#### Statistical analysis

Differences in immunoblots and immunofluorescence were assessed using t-test. The neuronal and vascular data were analyzed using linear mixed effects (lme function in nlme package, R) with subject as random effect to account for between-animal variations. This modeling yields sensible restricted maximum likelihood estimates from unbalanced allocation of animals by factor^46^, presently resulting from attrition. The MI, TTP changes and flow change between normo- and hypercapnia were modeled as linear functions of genotype. In the TgF344-AD rats, the penetrating vessel’s TTP change with hypercapnia was modeled as a linear function of the vessel’s amyloid load. We did not observe any sex-differences in our statistical analyses of all readout measures; thus the data was collapsed for all analyses.

### Electrophysiology

Four TgF344-AD and 4 age-matched nTg rats were used for the electrophysiological recordings. Surgery was performed under isoflurane (5% for induction and 2% for maintenance). After an intravenous catheter was inserted in the tail vein, the skin covering the skull and dura mater were removed and a cranial window over the somatosensory cortex was prepared. A two-shank linear multielectrode array (LMA) was lowered into the cortex. Each shank was equipped with two platinum/iridium recording sites (200 μm in diameter) which are spaced by 0.4 mm (Microprobes for LifeScience). At the end of the surgery, the isoflurane was gradually lowered and the animals were induced into a light sedative state by a bolus of propofol (7.5 mg/kg) followed by continuous intravenous administration of propofol at 44 mg/kg/hr via the tail vein. Resting state local field potentials were amplified (AM-systems 3600) between 0.3 Hz and 5 kHz, sampled at 20 kHz (DataWave) and stored on a desktop computer for off-line analysis.

Raw signals were filtered with a low-pass FIR filter with 500 Hz cutoff and two minutes of continuous recordings used for subsequent analysis. The modulation index (MI) was estimated with Matlab Toolbox by Onslow^47^ (https://www.cs.bris.ac.uk/Research/MachineLearning/pac/). To estimate PAC, we adopted the method presented by Canolty^48^, whereby the high frequency envelope amplitude and the low frequency instantaneous phase comprise the amplitude and phase of the complex valued composite signal. A population of 50 shuffled signals were created and compared to the original signal so as to generate a distribution of MI values. MI values lying in the top 5% of this distribution (after Bonferroni correction) were deemed significant. Theta band (4–8 Hz) was divided into four 1 Hz bins, while gamma band (30–140 Hz) was subdivided into 4 Hz bins.

## Results

### AD Pathology

**Figure 1:**
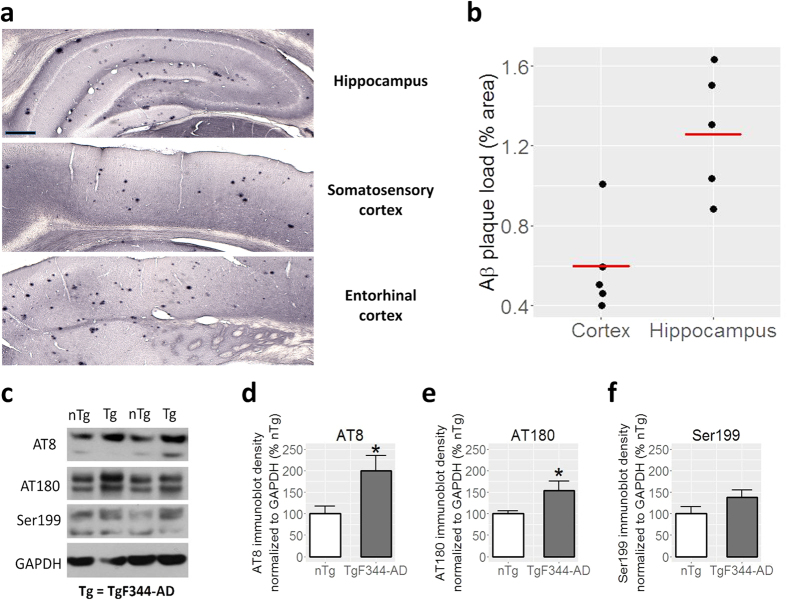
Characterization of Aβ plaques and tau hyperphosphorylation. (**a**) Representative micrographs of hippocampus (top), somatosensory cortex (middle) and entorhinal cortex (bottom) labeled with 6F3D showing deposition of Aβ plaques. Scale bar = 400 μm. (**b**) Percentage of cortical and hippocampal areas covered by Aβ plaques; n = 5. (**c**) Representative immunoblots showing that various phosphorylation sites of tau have increased levels in TgF344-AD rats compared to nTg controls including (**d**) clone AT8 which targets Ser202 and Thr205 (p = 0.030), (**e**) clone AT180 which targets Thr231 (p = 0.035), and (**f**) Ser199 (p = 0.13). n = 6. Bars represent mean + SEM. *p < 0.05. Full length blots are included in Supplementary Fig. 1a.

To characterize the AD pathology of the TgF344-AD cohort, we analyzed the Aβ plaque load and tau hyperphosphorylation of 9 month-old TgF344-AD rats. Quantitative histological analysis of cortical and hippocampal regions showed 0.6 ± 0.1% (mean ± SEM) and 1.3 ± 0.1% Aβ plaque coverage, respectively (Fig. 1a,b). We found the distribution of plaques across the cortex non-uniform, with greater plaque load in the insular, piriform and entorhinal cortices and fewer plaques in all other cortical regions. These results fall within the Aβ progression arc originally reported by Cohen and colleagues^38^, who detected no amyloid pathology in the cingulate cortex and hippocampus at 6 months of age, yet observed percent area covered with plaques of ~2.5% in the cingulate cortex and ~3% in the hippocampus at 16 months of age. While phosphorylation of tau is a normal process required for regulation of tau binding to microtubules, abnormal phosphorylation, termed hyperphosphorylation, results in paired helical filaments that in turn form NFTs, a pathological hallmark of AD^49^,^50^. Of the 21 phosphorylated sites thus far identified, we examined for Ser202 and Thr205 with AT8 anti-phosphorylated tau^51^; Thr231 with AT180 anti-phosphorylated tau^52^; and Ser199 with Ser199 anti-phosphorylated tau^53^,^54^ as these sites have been shown to be useful for identifying early stage of tauopathy in AD^55^. While the level of phosphorylation at Ser199 in our TgF344-AD rats was not significantly different from the nTg cohort (p = 0.13), the phosphorylation of tau at Ser202/Thr205 (p = 0.030) was increased by a factor of two; and the phosphorylation of tau at Thr231 (p = 0.035) was increased by a factor of 1.5 compared to nTg rats, demonstrating early tauopathy in the transgenic cohort (Fig. 1). These results are consistent with the earlier study in this model^38^, which reported increased tau hyperphosphorylation as early as 6 months of age; however, a direct comparison is not possible due to differences in the panel of antibodies utilized in that *vs.* present study.

### Cerebral amyloidosis and vascular wall remodeling

**Figure 2:**
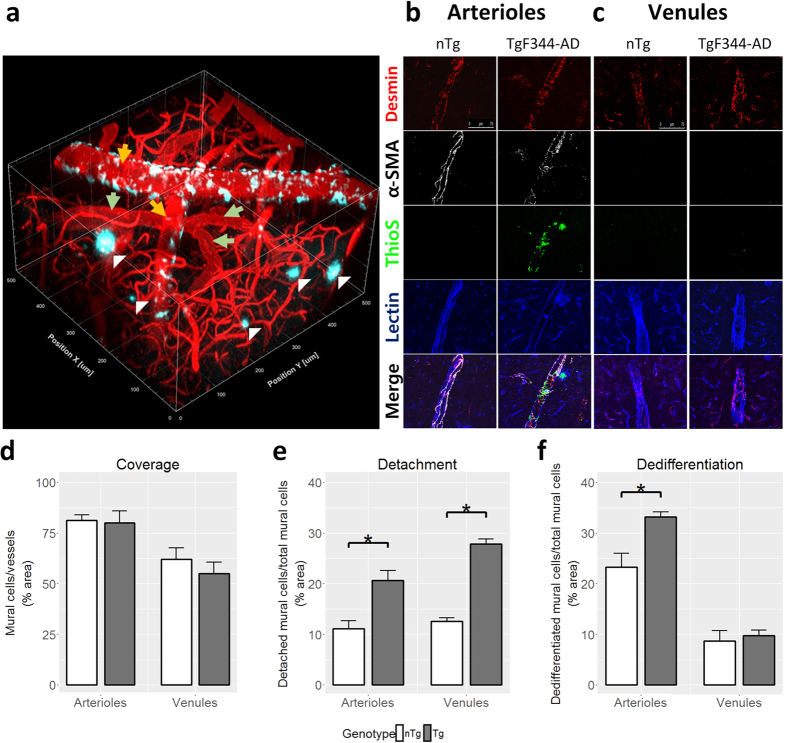
Morphology of the somatosensory cortical microvasculature. (**a**) 3D visualization of two-photon fluorescence images acquired in the somatosensory cortex of a 9-month-old TgF344-AD rat showing pial and penetrating arterioles (orange arrow) and venules (green arrow) with vascular amyloid deposits (cyan) and parenchymal plaque (white triangle). Representative immunofluorescence images of arterioles (**b**) and venules (**c**) from nTg and TgF344-AD rat, demonstrating vascular amyloid deposition. Scale bar 75 μm. (**d**) Percentage of vessel area covered by mural cells (n = 5). (**e**) Percentage of mural cells exhibiting detached morphology *vs.* total mural cells (n = 5). (**f**) Percentage of mural cells with a dedifferentiated morphology over total mural cells (n = 5). *p < 0.05.

**Figure 3:**
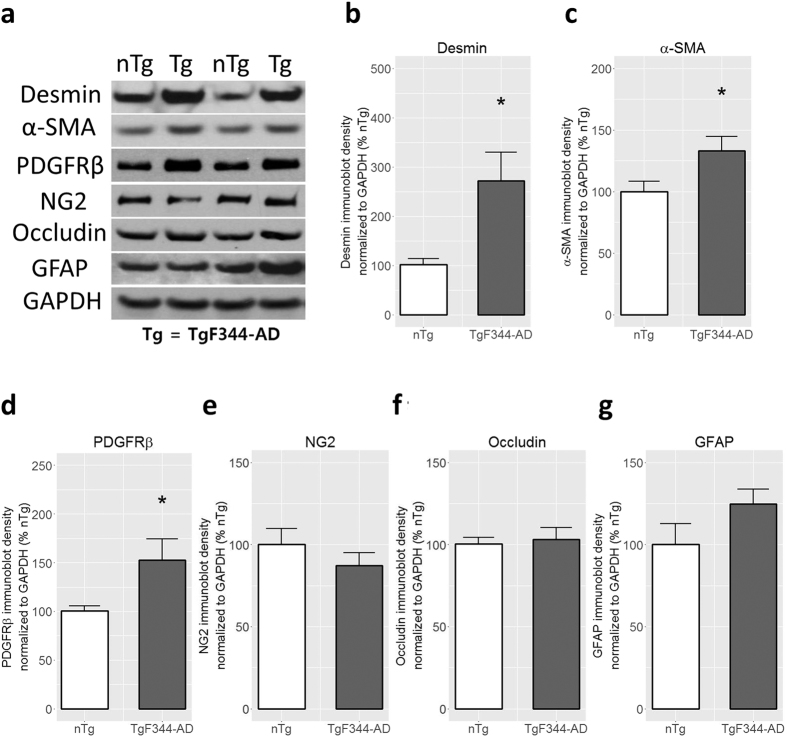
Immunoblot analysis of protein levels in cortical vessels. Desmin (**a** and **b**; p = 0.013), α-SMA (**a** and **c**; p = 0.035), and PDGFRβ (**a** and **d**; p = 0.034) expressions are significantly increased in the TgF344-AD rats compared to nTg rats. Expressions of NG2 (**a** and **e**), occludin (**a** and **f**), and GFAP (**a** and **g**) did not differ between nTg and TgF344-AD rats. Bars represent mean + SEM. *p < 0.05. Full length blots are included in Supplementary Fig. 1b.

Cerebral amyloidosis in TgF344-AD rats was previously reported to progress in an age-dependent manner from 6 to 16 months of age^38^. At 9 months of age, our TgF344-AD rats exhibited significant amount of vascular Aβ deposits on the cortical penetrating arterioles (Fig. 2a), with 39.0 ± 3.7% (mean ± SEM) of penetrating arteriolar walls covered by ThioS-positive Aβ aggregates (Fig. 2b). At this stage, the vascular amyloid was primarily localized to the cortical regions. Our previous work in an AD mouse model has shown that dysregulation of mural cells lining the walls of penetrating arterioles and venules is a prominent pathology associated with vascular Aβ deposition^16^. Here, we found that desmin-positive mural cells on penetrating vessels of TgF344-AD rats were morphologically different from those of nTg rats suggesting mural cell remodeling (Fig. 2b,c). Although TgF344-AD rats showed no loss of mural cell volume/coverage (Fig. 2d), the percentage of mural cells on both arterioles and venules that were detached from other mural cells was increased (Fig. 2e). Arteriolar mural cells additionally displayed an increased number of cells morphologically distinct from striated morphology seen in most arteriolar-associated mural cells, indicative of activation/dedifferentiation (Fig. 2f). To confirm this activation, we measured the protein expressions of several mural cell markers including desmin, α-SMA, PDGFRβ, and NG2 (Fig. 3a–e). The cytoskeletal proteins desmin and α-SMA were upregulated in TgF344-AD rats in comparison to nTg rats (Fig. 3a–c). PDGFRβ, which mediates key signaling pathways in mural cells, was also upregulated in TgF344-AD rats (Fig. 3a,d) indicating that mural cells may be starting to undergo structural remodeling. As mural cells are integral components of the blood-brain barrier (BBB), we examined whether structural proteins of the BBB were affected as a result of mural cell remodeling. However, we did not find significant differences between TgF344-AD and nTg rats with regard to expressions of either occludin or GFAP, both markers of BBB integrity (Fig. 3a,f,g).

### Vascular dysfunction

**Figure 4:**
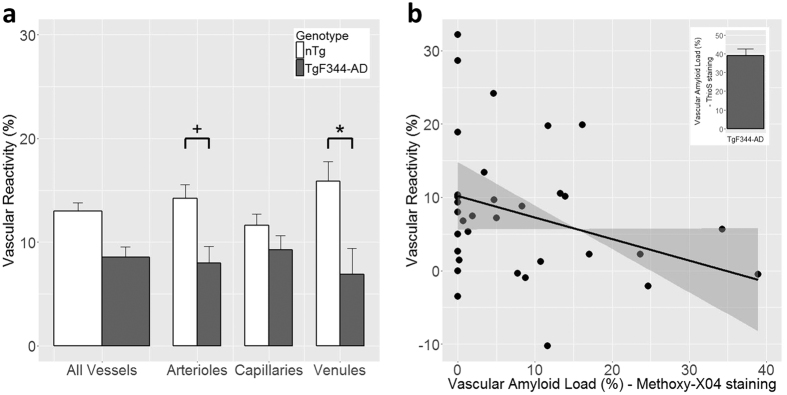
Vascular reactivity compromise in TgF344-AD rat and its dependence on the vessel’s amyloid load. (**a**) Bar plot (mean + SEM) of vascular reactivity in each vessel type: vascular reactivity of arterioles (+p = 0.056) and venules (*p < 0.05) was lower in TgF344-AD than in nTg rats. (**b**) The scatter plot of vascular reactivity *vs.* vascular amyloid load of penetrating arterioles estimated from the methoxy-X04 staining of vascular amyloid along with the linear regression to the data: the vessel’s reactivity decreases with increasing amyloid load, with the slope of the linear regression of −0.29 ± 0.15 (mean ± SEM; p = 0.065; R^2^ = 0.26). The grey shaded ribbon shows the 95% confidence interval on the fit. On average, 39.0 ± 3.7% (mean ± SEM) of penetrating arteriolar walls were covered by ThioS-positive Aβ aggregates.

We next set out to investigate the effect of progressive amyloidosis on cortical microvasculature in this model of AD. Two-photon fluorescence microscopy was employed to visualize superficial microvascular network along with the parenchymal plaques and vascular amyloid on pial and penetrating vessels in the somatosensory cortex from the pial surface to 450 μm into the cortex (Fig. 2a). (Of note, amyloid deposition varies both across brain regions and across cortical layers). On visual inspection, vascular amyloid was preponderantly, though not exclusively, deposited on arterioles. The vascular amyloid load of individual vessels was quantified from the 3D microvascular network segmentation as a ratio of surface area of amyloid to the surface area of the penetrating portion of the corresponding vessel. The superficial vascular amyloid load thus estimated from the segmentation of *in vivo* images of the 3D microvascular network (10.5 ± 2.5% (mean ± SEM)) was lower than that measured by ThioS staining (39.0 ± 3.7%) of brain sections (Fig. 4b). The lower abundance of vascular amyloid in the first 500 μm below the cortical surface in comparison to the rest of the cortex was confirmed by qualitative visual inspection of the brain sections.

Cerebrovascular reactivity reflects dilatory capacity of cerebral vasculature. In the current study, the cerebrovascular reactivity was quantified as the difference in TTP (i.e. time interval between the injection of the bolus and the time of peak fluorescent signal intensity in the vessel) between normo- and hypercapnic conditions, normalized by the TTP during normocapnia. Under physiological conditions, hypercapnia induces cerebral blood flow increase so that TTP shortens^56^. We first contrasted the hypercapnic response of nTg and TgF344-AD rats in each vessel type (Fig. 4a). The TgF344-AD rats’ arterioles and venules respectively showed 44% (TgF344-AD: 8.0 ± 1.8% (mean ± SEM), N = 7 vs. nTg: 15 ± 2.7%, N = 9; p = 0.056) and 51% (TgF344-AD: 7.3 ± 2.4%, N = 6 vs. nTg: 15 ± 2.8%, N = 8; p = 0.044) lower reactivity than did those from nTg rats. No attenuation of the vascular reactivity was observed in the capillaries. The average reactivity in each type of vessel for both cohorts is listed in Table 1. Next, we examined the correlation between the vascular reactivity of the penetrating vessels and their amyloid load using data from 33 penetrating arterioles (Fig. 4b). The linear regression had a slope of −0.29 ± 0.15 (mean ± SEM; p = 0.065; R^2^ = 0.26), demonstrating a strong trend toward attenuation of vessels’ reactivity to hypercapnia with increasing vascular amyloid load.

### Nulling of vascular network flow increase to hypercapnia

**Figure 5:**
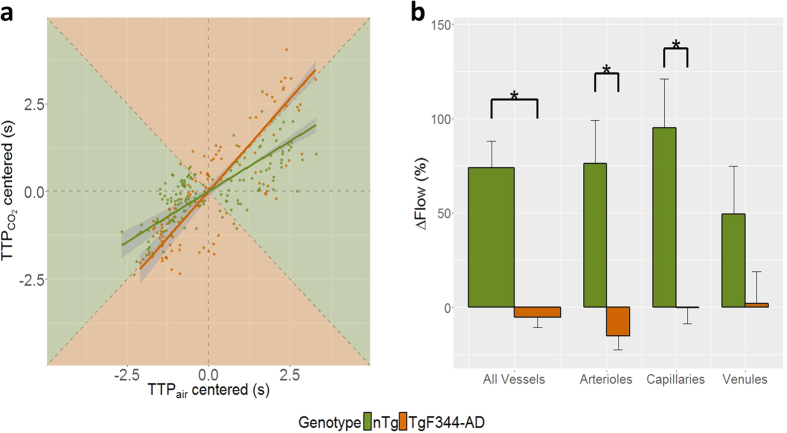
Microvascular flow change in response to hypercapnia. (**a**) A scatter plot of mean-centered TTP during hypercapnia *vs.* during normocapnia in nTg (olive-green) and TgF344-AD (pumpkin) rats is shown, along with orthogonal distance linear regression fit with 95% confidence intervals on the fit indicated by the grey shaded ribbons. Flow change in response to hypercapnia is estimated by the inverse of the slope of the regression line. The background of the scatter plot is color-coded to signify the regions of flow rise (olive-green shading) or decline (pumpkin shading). The boundaries between the two shaded regions represent a slope of 1, i.e., no change in flow with hypercapnia. Hypercapnia-elicited flow change, relative to the baseline flow during normocapnia, is plotted in (**b**) where bars represent mean ± SEM. Whereas nTg rats showed an increase in flow with hypercapnia in all vessel types, there was no increase in flow in the TgF344-AD rats. Arteriolar and capillary flow responses were significantly smaller in TgF344-AD than in nTg rats. *p < 0.05. The estimated flow changes are listed in Table 2.

Another measure of vascular function that can be derived from bolus tracking data is the change in microvascular network blood flow with hypercapnia. The decrease in blood flow response to stimulation is commonly found in neurodegenerative diseases including AD^15^,^16^,^32^. Stimulation-induced decrease in mean of microvascular transit times coupled with a relatively larger decrease in dispersion of microvascular transit time has been reported and is considered an adaption mechanism to ensure effective delivery of oxygen during blood flow increases^43^,^57^,^58^,^59^. In contrast, lack of stimulation-induced transit time homogenization is thought to be present in various forms of neurodegeneration, including AD^57^, potentially contributing to transient hypoxia during heightened metabolic demands. We have previously exploited the stimulation-induced decrease in dispersion of microvascular transit times to estimate the flow response to hypercapnia^16^. Accordingly, Fig. 5a shows the scatter plot of mean-centered TTP values (hence subtracting the mean value so as to illustrate differences in slopes) across all vessels (147 in nTg and 117 in TgF344-AD rats) during hypercapnia *vs*. those during air breathing. The orthogonal distance linear regression lines are shown for nTg rats in olive-green and for TgF344-AD rats in orange. The network flow change is estimated by the inverse of the slope of the regression line, with the slope below 1 indicating reduced dispersion of TTP in response to hypercapnia and thus increase in flow, and vice versa. The plot background is shaded to aid in visualization of the flow change, with the olive-green shaded region corresponding to flow increases and the orange shaded region corresponding to flow decreases. As listed in Table 2, nTg rats’ vessels showed a hypercapnia-elicited increase in flow by 74 ± 14% (mean ± SEM), with largest flow increase seen in the capillaries (95 ± 26%). Contrastingly, TgF344-AD rats exhibited no change in flow in response to hypercapnia (−5 ± 5%). Further, the hypercapnia-induced flow change in arterioles and capillaries of TgF344-AD were significantly smaller than the corresponding changes in arterioles and capillaries of nTg animals (p = 1 × 10^−5^ and p = 1 × 10^−5^, respectively). Mean flow changes to hypercapnia, expressed in percentages of the baseline flow, are displayed in Fig. 5b. TgF344-AD rats in the early stage of AD progression exhibited pronounced vascular dysfunction, on the whole being unable to regulate network blood flow in response to hypercapnic stimulation.

### Neuronal network dysfunction

**Figure 6:**
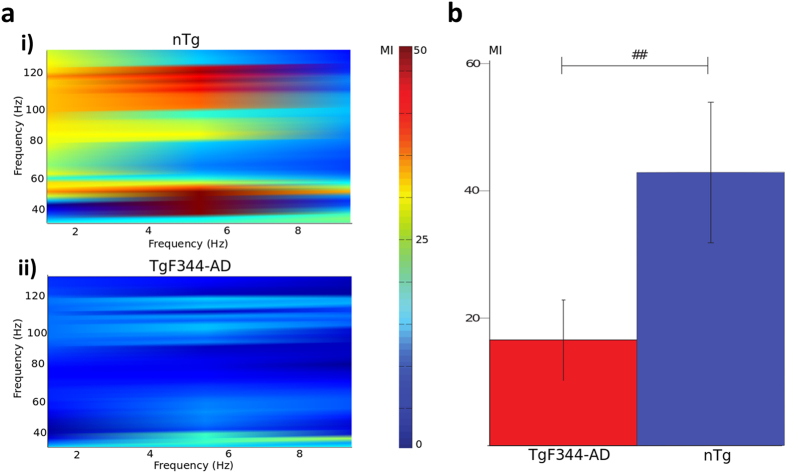
Phase amplitude coupling. (**a**) Modulation index in the neocortex of representative i) nTg and ii) TgF344-AD rats. Higher MI values are encoded as warmer colors. Theta-gamma modulation is present both in the low gamma (35–60 Hz, maxima of the modulating frequency 5.6 Hz for the nTg and 7.9 Hz for the TgF344-AD) and in the high gamma (100–120 Hz, maxima of the modulating frequency 5.8 Hz for the nTg and 6.1 Hz for the TgF344-AD) bands in the nTg animal and much attenuated in the TgF344-AD littermate. (**b**) Population bar plot of the average MI (across all electrodes of all animals within each cohort) showing a decreased MI in the TgF344-AD cohort (red) vs. nTg group (blue). ^##^p < 0.05.

It has been shown that theta and gamma band power level per se do not provide contrast between APP-deficient and wild-type mice even in the presence of Aβ accumulation and cognitive deficits^60^. However, accumulating evidence indicates that a more sensitive predictor of cognitive function is the coupling between the phase of (slow) theta oscillation and the amplitude of (faster) gamma oscillation because of its role in information processing^61^,^62^,^63^. PAC is one of the manifestations of cross-frequency coupling exhibited by neuronal network, whereby the phase of a lower frequency band modulates the amplitude of a higher-frequency band. Such coupling has been observed in various studies and is prevalent in humans^48^,^64^ and rodents^65^. Even though the cumulative effects of the excitatory and inhibitory signal interactions are incompletely understood, PAC has been shown to reflect temporal coordination of neuronal networks across or within brain regions^48^,^64^,^65^. We focused on the modulation index (MI) specifying the modulation that theta band (4–8 Hz) exerts on gamma band (30–140 Hz), as its importance has been shown in many studies of human neocortex^48^,^61^ and in mouse models of AD^60^,^66^. In particular, the alteration in theta-gamma PAC was demonstrated in one-month old TgCRND8 mice prior to Aβ accumulation, suggesting that theta-gamma PAC may be an early marker of AD-associated neuronal network dysfunction^66^. Figure 6a shows the theta-gamma PAC in a representative TgF344-AD animal and in an nTg littermate. Figure 6b summarizes the cohort-wise MI data. The average MI estimated in the somatosensory cortex of TgF344-AD rats (18.1 ± 4.9 (mean ± SEM)) was significantly reduced (p = 0.029) when compared to that of nTg littermates (45.2 ± 8.9), indicating an impaired functional interaction between the theta and the gamma bands in the transgenic cohort (Fig. 6b).

## Discussion

Altogether, our data show that TgF344-AD rats exhibit significant cerebrovascular and neuronal network dysfunction in the early stage of tau and Aβ pathologies. These findings underscore the complex and multifactorial nature of AD pathology, supporting the development of therapeutic interventions targeted at preemption and restoration of cerebrovascular and neuronal network function in early stage of AD.

The level of tau hyperphosphorylation in our study is in line with the tau hyperphosphorylation demonstrated by Cohen and colleagues in the same TgF344-AD rat model^38^ (and followed by NFT formation at 16 months of age^38^) and in human AD brain^50^,^51^,^67^. Further, the sites of hyperphosphorylation on tau in our TgF344-AD rats match with those shown to exhibit pronounced phosphorylation in the early stage of AD^51^,^52^,^53^,^54^. While hyperphosphorylation of tau leads to tau aggregation and NFT formation^68^, the mechanistic understanding of the effect of tauopathy progression on the neuronal network functioning is incomplete^35^.

Present data also demonstrated the activation of mural cells in both arterioles and venules in early stage of disease progression in TgF344-AD rats, potentially signifying onset of vascular remodeling. Contrary to the venular mural cell degeneration observed previously in TgCRND8 mouse model in mid-stage of AD^16^, neither arterioles nor venules of TgF344-AD rats exhibited significant degeneration of mural cells as assessed using desmin, α-SMA, PDGFRβ, or NG2 at this stage of disease progression. The overexpression of PS1ΔE9 is known to cause modulation of the active site on γ-secretase, catalyzing not only the increase in the ratio of Aβ42 to Aβ40 through increased production of Aβ42 and decreased production of Aβ40^69^, but also cleavage of type-I transmembrane proteins such as Notch receptor, E-cadherin, N-cadherin, and VEGF, which are known to be involved in the regulation of angiogenesis^70^,^71^. However, the details of the mechanism of action of PS1ΔE9 on cerebrovascular integrity including angiogenesis are not known^70^.

Despite the absence of mural cell loss in the microvasculature, TgF344-AD rats in the early stage of AD-like progression exhibited significant cerebrovascular dysfunction. Present characterization of the relationship between arteriole’s amyloid load and its function expands the mechanistic understanding of microvascular dysfunction in AD. Penetrating vessels laden with Aβ are seen in as many as 90% of autopsy cases of AD patients^72^, where significant amount of smooth muscle cells in the tunica media of the vessel wall is replaced with Aβ deposits, debilitating vessel contractility^73^. In the present study, the reactivity of cortical penetrating arteriole to hypercapnia decreased with increasing amyloid load on the vessel walls. Earlier study by van Dijk and colleagues reported positive correlation between the vascular dysfunction and soluble Aβ in volunteers in presymptomatic stage of AD^74^; the authors attributed the increasing severity of vascular dysfunction to higher level of soluble Aβ resulting in increased level of vascular amyloid deposition and the loss of vascular contractility^73^,^75^. Our data provide direct evidence that in the early stage of AD pathophysiology, the severity of arteriolar dysfunction relates to amyloid coverage of the arteriole.

The functional impairment of penetrating arterioles observed in the current study is particularly significant in light of the critical role the cortical penetrating arterioles play in supplying blood to the cortex^76^. Each penetrating arteriole distributes blood to tissue within 350 μm radius in the rat cortex^76^. The failure of penetrating arterioles may thus well lead to episodes of ischemia/hypoxia during elevated tissue metabolism^76^,^77^. On the venular side, results from APP/PS1/Cx3cr1 mice in early stage of the disease revealed that small Aβ aggregates first appear in venous network, prior to the formation of vascular amyloid deposits on arterial walls^78^. Furthermore, it has been reported that in the course of dementia, venular mural cells are replaced with collagen^79^, with occlusive venular collagenosis leading to venous insufficiency and vasogenic edema^80^. The current observation of attenuated venular reactivity to hypercapnia in early stage of AD expands upon these findings on pathophysiology of draining vasculature, of particular significance in AD due to the importance of the paravenous pathway for the clearance of vascular amyloid^81^.

The vascular dysfunction in the absence of structural degeneration observed in this study suggests that there exist other pathological drivers of dysfunction not mediated by morphology. A large body of evidence identifies vascular dysfunction as a major predictor of AD development, yet the exact events that lead to vascular dysfunction and influence of vascular dysfunction on later cognitive decline are not completely understood^19^,^82^,^83^. Vascular dysfunction has strong correlation with the level of soluble Aβ species, more so than does the amyloidosis-induced vascular wall degeneration^32^,^84^,^85^. Adverse influences of soluble Aβ species, including diminished vasomotor responses of penetrating arterioles to vasoactive mediators such as ATP^85^ and mitochondrial dysfunction^86^, lead to increased production of reactive oxygen species and reduced Aβ clearance^85^,^86^ and can have direct impact on the vascular function prior to any cerebrovascular dysmorphology. Furthermore, the rise in soluble Aβ levels have been implicated in neuronal network alterations including tau hyperphosphorylation^87^ and synaptic dysfunction^88^,^89^, demonstrating the neurotoxic effect of soluble Aβ species.

In concert with the vascular dysfunction, TgF344-AD rats exhibited impaired theta-gamma PAC, indicating neuronal network dysfunction. The reduction of MI in TgF344-AD rats compared to nTg littermates is in line with attenuated theta-gamma coupling observed in a mouse model of AD during active walking^60^. In the present study cross-frequency coupling was estimated within the neocortex, but cross-frequency coupling has also been observed across functionally related brain regions. For example, hippocampalestriatal cross-frequency coupling is dynamically modulated alongside behavioral task demands in rat^90^, and hippocampal theta phase can also modulate neocortical gamma power^91^. The investigation of cross-frequency coupling within and between those areas in TgF344 rat model of AD requires further work. Cross-frequency coupling reflects the temporal coordination of neuronal networks across different brain regions, though the basic features of excitatory and inhibitory network connectivity that give rise to cross-frequency coupling of different frequencies and in different anatomical regions have not yet been defined.

As more potential treatments for AD focus on targeting early markers of disease^92^,^93^, identification of the link between early neurovascular network dysfunction and subsequent cognitive decline rises in importance. Studies to date suggest that the cognitive decline in AD can be traced to the neuronal network dysfunction^94^,^95^ and increased levels of soluble Aβ species^82^ in the early stage of disease. Also, vascular dysfunction in key brain regions involved in object recognition and a strong correlation between the degree of vascular dysfunction and the degree of memory impairment in individuals with high risk of AD suggest a contribution of vascular dysfunction to cognitive decline in AD^96^,^97^. To put the present flow response abolishment in context, behavioral testing of mice following pharmacological inhibition of endothelial and astrocytic vasoactive factors that resulted in 60% decrease in CBF response to whisker stimulation was associated with deficits in spatial memory, learning, object recognition, and sensorimotor functions, suggesting that the cerebrovascular dysfunction strongly influences cognitive performance^98^.

This study provides the first characterization of neurovascular dysfunction in early stage of pathology in the TgF344-AD rat model that recapitulates a broad spectrum of AD-like pathologies. In conjunction with further amyloidosis and tauopathy seen with the progression of the disease, the observed neurovascular deficits are expected to accelerate the progression of neuronal dysfunction and degeneration, as reported in the later stage of the disease in both animal models and human patients^19^,^36^,^38^. Future work will investigate the role of neurovascular dysfunction in cognitive decline and its reliability as a predictor of AD onset and progression. This will be a major step in the development of early-stage combinatorial interventional targets, of interest for effective treatment of the majority of AD patients, who exhibit a significant neurovascular pathology.

## Additional Information

**How to cite this article**: Joo, I. L. *et al*. Early neurovascular dysfunction in a transgenic rat model of Alzheimer’s disease. *Sci. Rep.* **7**, 46427; doi: 10.1038/srep46427 (2017).

**Publisher's note:** Springer Nature remains neutral with regard to jurisdictional claims in published maps and institutional affiliations.

## Supplementary Material

Supplementary Figure 1

## Figures and Tables

**Table 1:** Vascular reactivity to hypercapnia.

	Vascular Reactivity [%]		p-value
	nTg	TgF344-AD	
ALL	14 ± 1.6	7.9 ± 1.3	0.13
Arterioles	15 ± 2.7	8.0 ± 1.8	0.056
Capillaries	13 ± 2.9	8.4 ± 2.8	0.42
Venules	15 ± 2.8	7.3 ± 2.4	0.044

The vascular reactivity, or the magnitude of the percent change in TTP during hypercapnia relative to air, is listed for each vessel category as mean ± SEM.

**Table 2:** Microvascular flow change induced by hypercapnia.

	Flow Change (%)		p-value
	nTg	TgF344-AD	
ALL	74 ± 14	−5.3 ± 5.4	2.6 × 10^–10^
Arterioles	76 ± 23	−15 ± 7.5	1.7 × 10^−6^
Capillaries	95 ± 26	0 ± 8.5	7.2 × 10^−6^
Venules	49 ± 25	2.0 ± 17	0.12

Flow changes are reported as mean ± SEM.
