# Supplementary figures and images for: Early neurovascular dysfunction in a transgenic rat model of Alzheimer’s disease
Source: Sci Rep. 2017 Apr 12;7:46427. doi: 10.1038/srep46427 (PMC5388880; doi:10.1038/srep46427)

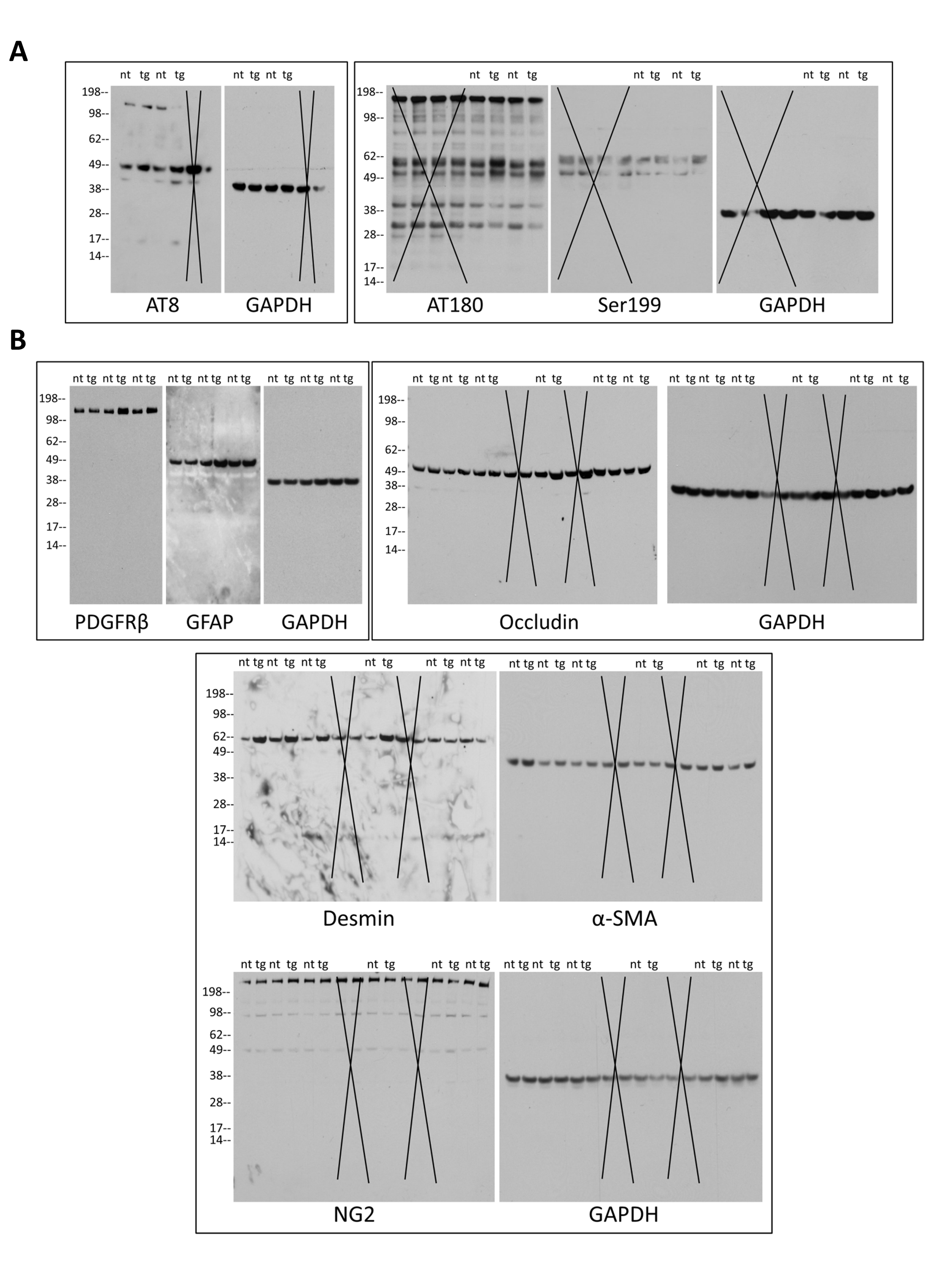

Supplement: Supplementary Figure 1 [file srep46427-s1.tiff]
